# Supplementary material for: Daytime eyeshine contributes to pupil camouflage in a cryptobenthic marine fish
Source: Sci Rep. 2018 May 9;8:7368. doi: 10.1038/s41598-018-25599-y (PMC5943592; doi:10.1038/s41598-018-25599-y)
Supplement: Supplementary file 1 — Supplementary information [file 41598_2018_25599_MOESM1_ESM.pdf]

Daytime eyeshine contributes to pupil camouflage in a cryptobenthic marine fish

Matteo Santon[1*], Pierre-Paul Bitton[1], Ulrike K. Harant[1], Nico K. Michiels[1]

[1] Animal Evolutionary Ecology, Institute for Evolution and Ecology, Department of Biology, Faculty of Science, University of Tübingen, Auf der Morgenstelle 28, 72076 Tübingen, Germany

*Corresponding author

Matteo Santon

Animal Evolutionary Ecology, Institute for Evolution and Ecology, Department of Biology, Faculty of Science, University of Tübingen, Auf der Morgenstelle 28, 72076 Tübingen, Germany

matteo.santon@uni-tuebingen.de

Supplementary information

Figure S1. Standards used in the field to estimate ambient and dark model pupil radiance. One upward-facing white PTFE diffuse reflectance standard as a proxy for down-welling light (right), two observer-facing PTFE standards as a proxy for side-welling light, one shaded (left) and one exposed (top centre) and a dark hole in a black block of PVC filled with black cloth to simulate the pupil of most other fishes (centre). Photo credit: M. Santon.

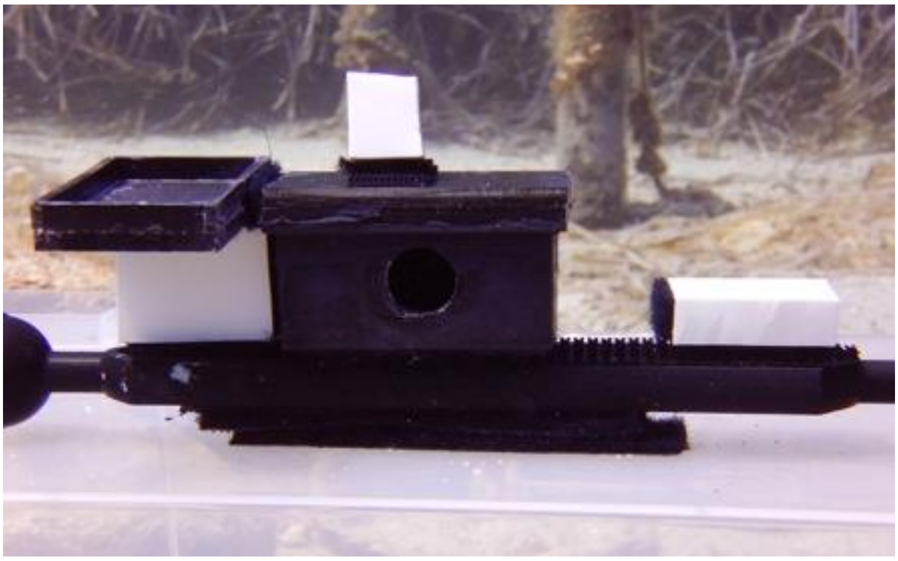

Table S1. Formulas used to estimate radiance. Mechanisms of eyeshine production and associate light field used to predict iris, natural pupil and dark model pupil radiance under three different light scenarios.

T: transmittance, R: reflectance, DW: down-welling light, SW: side-welling light.

DW and SW are approximated by measuring the radiance of a PTFE white standard parallel (DW) or perpendicular (SW) to the water surface.

Model	Natural Pupil	Dark control pupil	Iris
**Scorpionfish and triplefin exposed**	$DW_{exposed}$ x T_{PET} + SW_{shaded} x $R_{broadSAR}$	$SW_{exposed}$ x $R_{darkmodel}$	$SW_{exposed}$ x R_{iris}
**Only triplefin shaded**	$DW_{exposed}$ x T_{PET}	$SW_{exposed}$ x $R_{darkmodel}$	$SW_{exposed}$ x R_{iris}
**Only scorpionfish shaded**	SW_{shaded} x $R_{broadSAR}$	SW_{shaded} x $R_{darkmodel}$	SW_{shaded} x R_{iris}

Video S1. *S. porcus* daytime eyeshine. The pupil of *S. porcus* appears lit when the fish is exposed to sunlight, showing how daytime eyeshine could be used to conceal the otherwise dark pupil. Video credit: M Santon.
